# Toluene alters the intrinsic excitability and excitatory synaptic transmission of basolateral amygdala neurons

**DOI:** 10.3389/fnins.2024.1366216

**Published:** 2024-02-26

**Authors:** Kevin Braunscheidel, Michael Okas, John J. Woodward

**Affiliations:** Department of Neuroscience, Medical University of South Carolina, Charleston, SC, United States

**Keywords:** inhalants, basolateral amydgala, action potentials, slice electrophysiology, optogenetics, endocannabinoids

## Abstract

**Introduction:**

Inhalant abuse is an important health issue especially among children and adolescents who often encounter these agents in the home. Research into the neurobiological targets of inhalants has lagged behind that of other drugs such as alcohol and psychostimulants. However, studies from our lab and others have begun to reveal how inhalants such as the organic solvent toluene affect neurons in key addiction related areas of the brain including the ventral tegmental area, nucleus accumbens and medial prefrontal cortex. In the present study, we extend these findings and examine the effect of toluene on electrophysiological responses of pyramidal neurons in the basolateral amygdala BLA, a region important for generating emotional and reward based information needed to guide future behavior.

**Methods:**

Whole-cell patch-clamp electrophysiology recordings of BLA pyramidal neurons in rat brain slices were used to assess toluene effects on intrinsic excitability and excitatory glutamatergic synaptic transmission.

**Results:**

Acute application of 3 mM but not 0.3 mM toluene produced a small but significant (~20%) increase in current-evoked action potential (AP) firing that reversed following washout of the toluene containing solution. The change in firing during exposure to 3 mM toluene was accompanied by selective changes in AP parameters including reduced latency to first spike, increased AP rise time and decay and a reduction in the fast after-hyperpolization. To examine whether toluene also affects excitatory synaptic signaling, we expressed channelrhodopsin-2 in medial prefrontal cortex neurons and elicited synaptic currents in BLA neurons via light pulses. Toluene (3 mM) reduced light-evoked AMPA-mediated synaptic currents while a lower concentration (0.3 mM) had no effect. The toluene-induced reduction in AMPA-mediated BLA synaptic currents was prevented by the cannabinoid receptor-1 antagonist AM281.

**Discussion:**

These findings are the first to demonstrate effects of acute toluene on BLA pyramidal neurons and add to existing findings showing that abused inhalants such as toluene have significant effects on neurons in brain regions involved in natural and drug induced reward.

## Introduction

The amygdala plays a crucial role in regulating emotions, including the response to fear and anxiety (Andrewes and Jenkins, 2019) and is involved in aspects of substance and alcohol use disorders (Wassum and Izquierdo, 2015). For example, the amygdala mediates alcohol seeking behaviors (de Guglielmo et al., 2016), alcohol cue reactivity in individuals with alcohol use disorder (Claus et al., 2011), and anxiety-like behaviors during withdrawal from chronic alcohol use (Menzaghi et al., 1994; Diaz et al., 2011). In particular, the basolateral region of the amygdala (BLA) is involved in reinstatement of alcohol and drug seeking behavior (Keistler et al., 2017; Chesworth and Corbit, 2018; Pelloux et al., 2018) and shows alterations in neural activity following chronic alcohol or drug exposure (Lack et al., 2007; Marinelli et al., 2010; Corbett et al., 2023) including reduced sensitivity to acute ethanol (Robinson et al., 2016).

While the effects of alcohol and other drugs of abuse such as psychostimulants and opiates on BLA neuronal function have been extensively studied, little is known regarding the actions of abused inhalants on BLA activity. Inhalants are comprised of several classes of volatile agents including organic solvents such as toluene and are used for intoxicating purposes with a significant incidence of use among children and adolescents (Substance Abuse and Mental Health Services Administration (SAMHSA), 2021). An interaction between toluene and alcohol has been demonstrated in rodents (Pryor et al., 1985) as well as in humans with substance use disorder (Marin-Navarrete et al., 2016) suggesting common underlying neurobiology. In fact, toluene and alcohol have a similar chemical profile, overlapping pharmacology, as well as abuse potential (reviewed by Beckley and Woodward, 2013; Woodward and Braunscheidel, 2023). Despite this, few studies have assessed the effects of volatile organic solvents on the amygdala although one reported increased c-Fos immunoreactivity, a proxy for cellular activity, in the rat amygdala and other brain areas following a brief exposure to abuse-level concentrations of toluene vapor suggesting changes in BLA neural activity (Perit et al., 2012).

Previous studies from our laboratory using *ex vivo* slice electrophysiology revealed circuit and cell specific effects of toluene on the activity of mPFC projecting VTA dopamine neurons (Beckley et al., 2013), accumbens projecting mPFC neurons (Wayman and Woodward, 2018a,b) and D2 medium spiny neurons in the nucleus accumbens (Okas et al., 2023). Some of toluene’s inhibitory effects on glutamatergic synaptic transmission were shown to be mediated by a cannabinoid type 1 (CB1) receptor-dependent mechanism (Beckley and Woodward, 2011; Beckley et al., 2016). CB1 receptors are also expressed on presynaptic terminals in the BLA, and activation of CB1R, Gi-coupled signaling reduces neurotransmitter release (Melis et al., 2004; Busquets-Garcia et al., 2018). Given the important role of the BLA in emotional processing and reward learning and its sensitivity to various intoxicating substances, the present study examined the effects of acute toluene on the intrinsic excitability and glutamatergic transmission of BLA glutamatergic neurons.

## Materials and methods

### Animals

Male Sprague–Dawley rats (P77–P86 on arrival, Envigo RMS) were housed in pairs in polypropylene cages on a reverse light cycle (lights off at 09:00) in a climate-controlled room with food and water delivered *ad libitum*. For current clamp experiments, animals remained in their homecage until testing at age P105–P125. For voltage clamp experiments, animals underwent stereotaxic surgery 1 week after arrival for viral infusion of channelrhodopsin-2 (ChR2). Deep anesthesia was achieved via an isoflurane vaporizer (air flow 1 L/min, 5% induction, 2–3% maintenance) and 300 nL of AAV2-hSyn-ChR2-EYFP (AddGene) was injected into the prelimbic portion of the mPFC (AP: ±2.95; ML: ±0.6; DV: ±2.85 mm). Rodents were given 3–7 weeks of recovery to allow for channelrhodopsin-2 expression in mPFC in terminals of the BLA before recording at age P115–P145.

### Preparation of brain slices

As previously described (Wayman and Woodward, 2018b), brain tissue was rapidly removed and placed in an ice-cold sucrose solution that contained (in mM): sucrose (200), KCl (1.9), NaH_2_PO_4_ (1.4), CaCl_2_ (0.5), MgCl_2_ (6), glucose (10), ascorbic acid (0.4), and NaHCO_3_ (25); osmolarity 305–315 mOsm. This solution was bubbled with 95% O_2_/5% CO_2_ to maintain physiological pH. Sections containing the BLA were cut coronally into 300 μm slices using a Leica VT1000 vibrating microtome with a double walled chamber through which cooled solution (2°C–4°C) circulated (Isotemp 3006, Fisher Scientific). Slices were transferred to a warmed chamber (32°C–34°C) containing a carbogen-bubbled aCSF solution containing (in mM): NaCl (125), KCl (2.5), NaH_2_PO_4_ (1.4), CaCl_2_ (2), MgCl_2_ (1.3), glucose (10), ascorbic acid (0.4), and NaHCO_3_ (25); osmolarity 290–300 mOsm for 30 min, and then kept at room temperature in carbogen-bubbled aCSF for at least 45 min before recordings.

### *Ex vivo* electrophysiology

Brain slices were transferred to the recording chamber and perfused with oxygenated aCSF at a flow rate of 1.5 mL/min. The temperature was maintained at 34°C during the course of the recordings with in-line and bath heaters (Warner Instruments). A horizontal pipette puller (P-97, Sutter Instrument) was used to pull recording pipettes that were constructed from thin-walled borosilicate capillary glass tubing (I.D. 1.0 mm, O.D. 1.50 mm; Sutter Instruments). Pipettes were filled with an internal solution containing (in mM) the following: K-gluconate (120), HEPES (10), KCl (10), MgCl_2_ (2), Na_2_ATP (2), NaGTP (0.3), EGTA (1), a pH 7.3–7.4, osmolarity of 285–295 mOsm, and resistances ranging from 3 to 4 MΩ. BLA pyramidal neurons were visually identified using an Axioskop FS2 microscope using landmarks illustrated in a rat brain atlas (Paxinos and Watson, 1986). Following the formation of a gigaohm seal, light suction was applied to break through the cell membrane and achieve whole-cell access,. Neurons with an access resistance greater than 20 megaohm were not used for analysis. Recorded events were acquired with an Axon MultiClamp 700A (Molecular Devices), filtered at 4 kHz and digitized at a sampling rate of 10 kHz with an Instrutech ITC-18 analog-digital converter (HEKA Instruments) controlled by AxographX software (Axograph Scientific) running on a Macintosh G4 computer (Apple).

#### Intrinsic excitability

In order to study intrinsic excitability of BLA neurons, the resting membrane potential neurons was recorded under current clamp mode and then adjusted to ~ −70 mV for electrophysiological assessments of intrinsic excitability. A current ramp (0 to 500 pA over 1 s) was performed on each cell to determine rheobase. Then, action potentials were elicited using a 1 s pulse of current (rheobase +50 pA) at 0.1 Hz for 15 min (2 min baseline, 8 min treatment, 5 min washout). Internal resistance was calculated by measuring the voltage deflection in response to a 50 ms, 30 pA hyperpolarizing pulse given prior to each current pulse. Traces in which internal resistance deviated more than 25% from baseline were excluded from analysis. Recordings were analyzed offline for the number of spikes and action potential characteristics in response to each current step using AxographX software.

#### Glutamatergic synaptic transmission

Using a separate cohort of animals expressing channelrhodopsin-2 in mPFC neurons, voltage clamp experiments were performed to measure the effects of bath applied toluene on light-evoked synaptic glutamate transmission of BLA neurons. For these experiments, K-gluconate and KCl in the pipette solution were replaced with CsCl (120 mM). To isolate monosynaptic AMPA-mediated currents in BLA neurons, the extracellular recording solution also contained 250 nM tetrodotoxin (American Radiolabeled Chemicals, Inc.), 500 μM 4-aminopyridine (Sigma), and 50 μM AP5 (Tocris). In some experiments, 0.75 μM AM 281 (Tocris) was included in the bath solution to inhibit CB1 receptors. Following breakthrough, EPSCs were induced by photostimulation of mPFC terminals in the BLA via pulses of 470 nm LED light (LEDD1B, Thor Labs). Stimulus power ranged from 1.1–2.45 mW and generated EPSCs ranging from 100–400 pA. Traces were obtained during pairs of photostimulation pulses (1–5 ms with 150 ms inter pulse interval) and collected at a regular interval of 0.05 Hz for 15 min (2 min baseline, 8 min treatment, 5 min washout).

In all experiments, baseline values were collected until responses were stable (~1–5 min before recordings began). For toluene treatments, a known volume of HPLC grade toluene (Sigma-Aldrich, Saint Louis, MO) was added to pre-gassed aCSF and immediately perfused into recording bath using teflon tubing to minimize solvent loss. To control for loss of oxygen in the pre-gassed toluene solution, Sham recordings also used pre-gassed aCSF. Previous studies in our laboratory monitored the loss of toluene from experimental recording solutions and found that the concentration of toluene 15 min after dilution was 77.9 ± 15% (mean ± SEM) of baseline value obtained at 0 min (Cruz et al., 1998). Following this initial rapid loss because of volatility, toluene concentrations in recording solutions were relatively constant. Concentrations of toluene reported in the results section are not corrected for this loss. Once toluene was applied to a slice, no subsequent cells were recorded from that slice.

All procedures were performed in compliance with Medical University of South Carolina IACUC protocols in strict accordance with the NIH Guide for the Care and Use of Laboratory Animals.

### Data analysis

Data were analyzed with Prism 10 software (Graphpad Inc.) using a repeated measures mixed effects model with treatment and time as factors. Values during and following toluene exposure were compared to baseline with Dunnett’s post-hoc test. For secondary measures of intrinsic excitability, averaged responses in the last minute of treatment and washout were compared to baseline (repeated measures one-way ANOVA, Dunnett’s post-hoc).

## Results

### Toluene increases the intrinsic activity of BLA neurons

**Figure 1:**
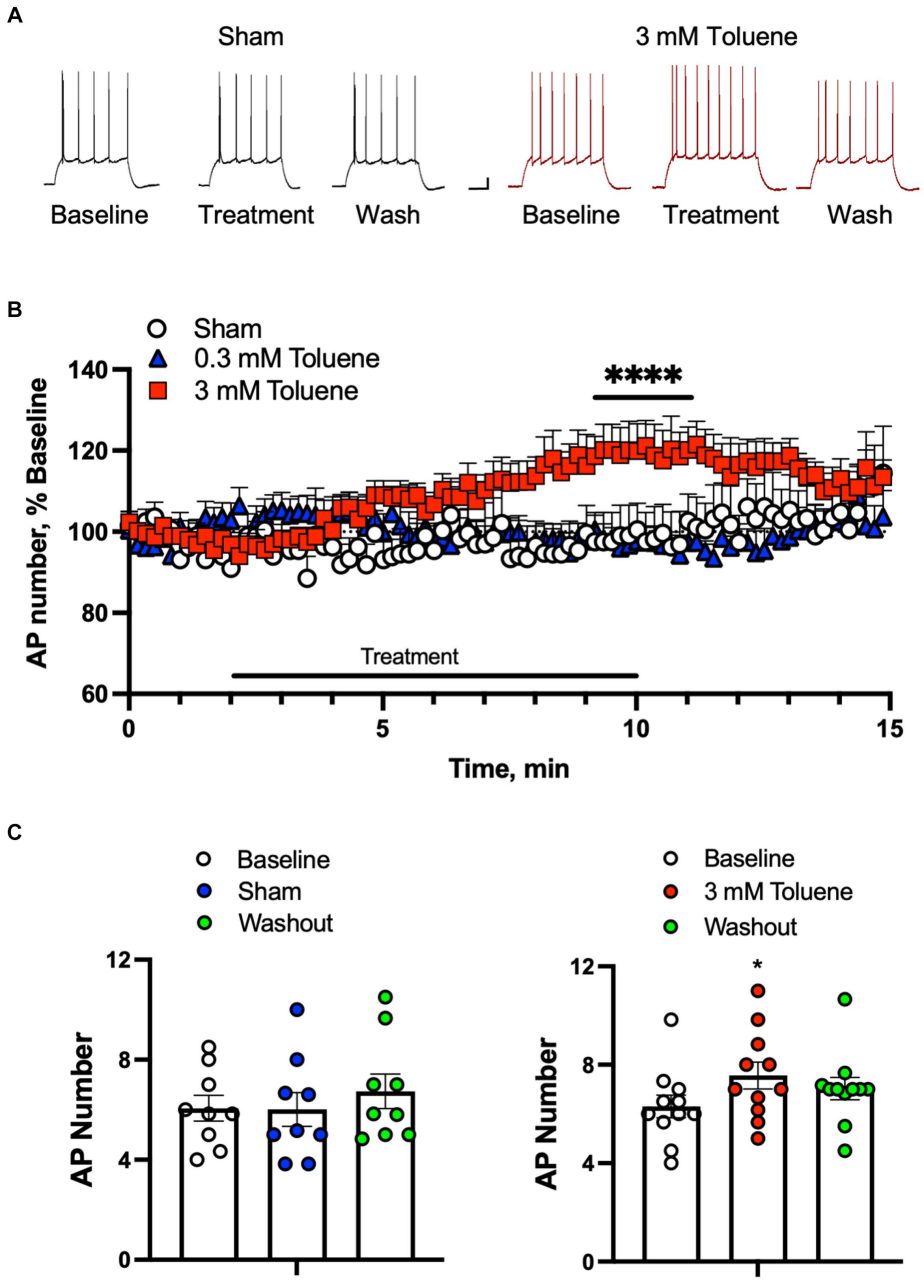
Toluene increases the intrinsic excitability of BLA pyramidal neurons. **(A)** Representative spike trains evoked by direct current injection in BLA pyramidal neurons under baseline, treatment, and washout of Sham aCSF (black, left) or 3 mM toluene (right, red). Scale bars: *x*-axis 100 msec, *y*-axis 10 mV. **(B)** Time course of number of evoked APs during exposure to Sham aCSF, 0.3 mM toluene or 3.0 mM toluene (expressed as percent of corresponding baseline). Symbol (****) indicates significant time × treatment interaction (*p* < 0.0001), repeated measures mixed effects model. **(C)** Summary of changes in action potential number before, during and after exposure to Sham aCSF (left panel) or 3.0 mM toluene (right panel). Symbol (*) indicates significant difference (*p* < 0.05) from baseline, one-way ANOVA with Dunnett’s post-hoc. Data are mean + SEM; aCSF Sham (*n* = 8 cells/3 animals); 0.3 mM toluene (*n* = 8 cells/5 animals), 3 mM toluene (*n* = 11 cells/4 animals).

In order to determine the effect of toluene on the intrinsic excitability of BLA neurons, action potentials (APs) were evoked by direct current injection designed to elicit 5–10 spikes (rheobase +50 pA) during baseline, treatment, and washout of aCSF, 0.3 or 3 mM toluene (Figures 1A,B). Each neuron was exposed to only one condition and the data were expressed as a percent of firing normalized to the 2 min baseline period (Figure 1B). Statistical analysis of the data shown in Figure 1B using a repeated measures mixed-effects model revealed no significant main effect of treatment (*F*_(2,24)_ = 2.65, *p* = 0.082) but a significant time x treatment interaction (*F*_(178,2127)_ = 2.57, *p* < 0.0001). To determine which data drove this interaction, we averaged the last two minutes of baseline, toluene exposure and washout for each neuron under each condition. There was no effect of Sham (Figure 1C, left panel; main effect of treatment *F*_(2,16)_ = 2.6, *p* = 0.10; one-way repeated measures ANOVA) or 0.3 mM toluene (*F*_(2,24)_ = −0.03, *p* > 0.99; One-way repeated measures ANOVA, data not shown) on BLA action potential firing. However, the number of action potentials was significantly increased during exposure to 3 mM toluene (Figure 1C, right panel; main effect of treatment, *F*_(2,20)_ = 7.39, *p* = 0.0039, one-way ANOVA; post-hoc Dunnett’s test, treatment vs. baseline, *q*_(20)_ = 3.83, *p* = 0.0020), and this effect reversed following washout (washout vs. baseline, *q*_(20)_ = 2.22, *p* = 0.070).

**Figure 2:**
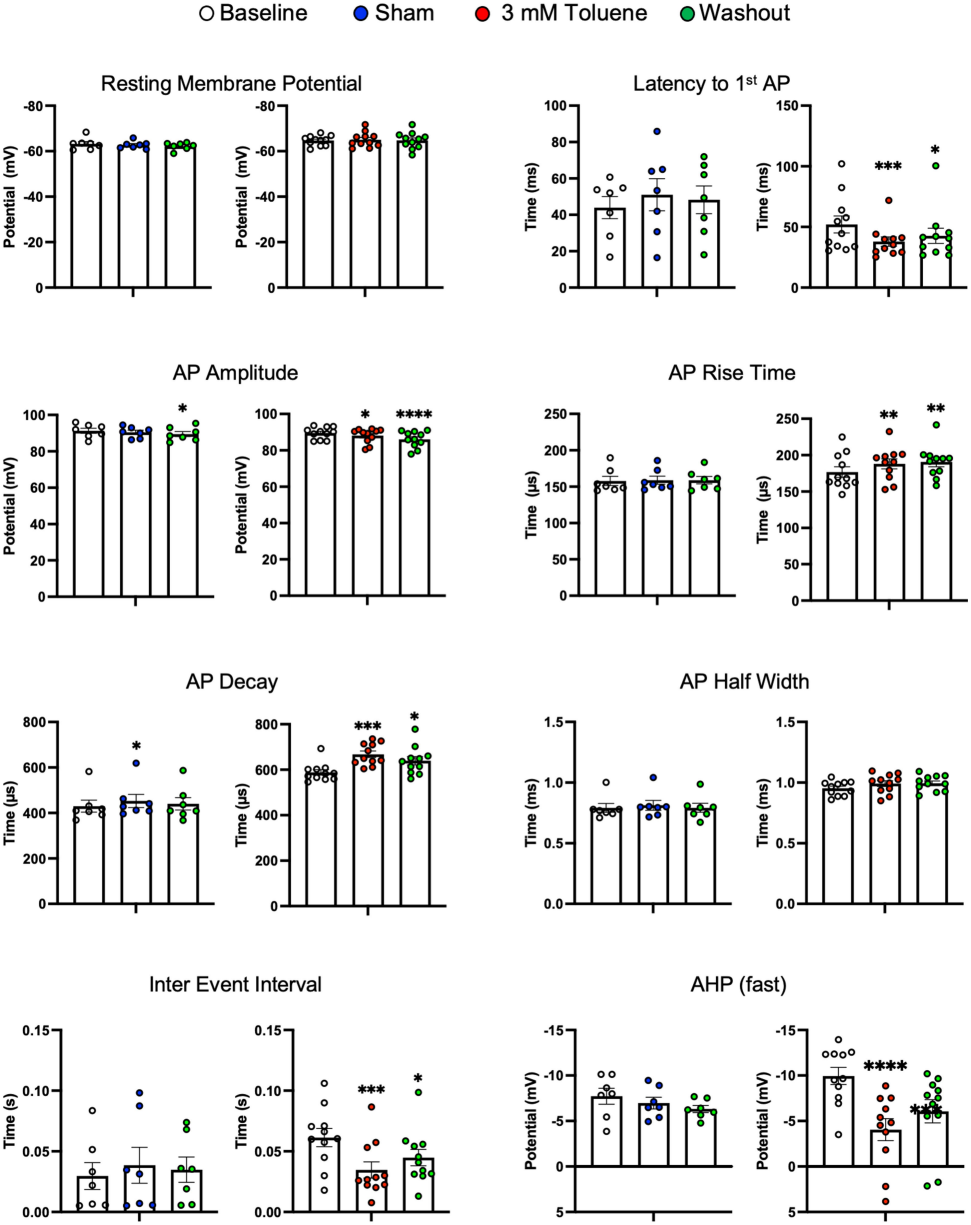
Summary of changes in electrophysiological parameters before, during and after exposure to Sham aCSF or 3.0 mM toluene. Graphs show resting membrane potential, latency to first action potential, AP amplitude, AP rise time, AP decay, AP half width, inter event interval, and fast after-hyperpolarization potential. Data are expressed as mean ± SEM; aCSF Sham (*n* = 8 cells/3 animals), toluene (*n* = 11 cells/4 animals). Symbols: ^*^p < 0.05, ^**^p < 0.01, ^***^p < 0.001, ^****^p < 0.0001; value significantly different from baseline, one-way ANOVA with Dunnett’s post-hoc.

Figure 2 shows that the increase in action potential firing during exposure to 3 mM toluene was accompanied by changes in several other measures including decreased latency to fire (vs. baseline *q*_(20)_ = 4.18, *p* = 0.0009), decreased AP amplitude (vs. baseline *q*_(20)_ = 2.39, *p* = 0.049), decreased inter-event interval (vs. baseline *q*_(20)_ = 4.45, *p* = 0.005), and a smaller fast after-hyperpolarization potential (vs. baseline *q*_(20)_ = 6.58, *p* < 0.0001). However, in the presence of 3 mM toluene, AP rise time increased (vs. baseline *q*_(20)_ = 3.27) and AP decay time increased (vs. baseline *q*_(20)_ = 4.79, *p* = 0.0002), that would be expected to decrease overall excitability. Sham aCSF treatment did not affect spike number, resting membrane potential, latency to fire, AP rise time, AP decay, AP half width, inter event interval, or fast afterhyperpolarization potential. However, a small, yet statistically significant decrease in AP amplitude was detected following washout of aCSF (vs. baseline *q*_(12)_ = 3.38, *p* = 0.0040) accompanied by an increase in AP decay observed during aCSF treatment (vs. baseline *q*_(12)_ = 2.65, *p* = 0.038).

### Toluene dose-dependently inhibits excitatory mPFC-BLA signaling in a CB1R-dependent manner

**Figure 3:**
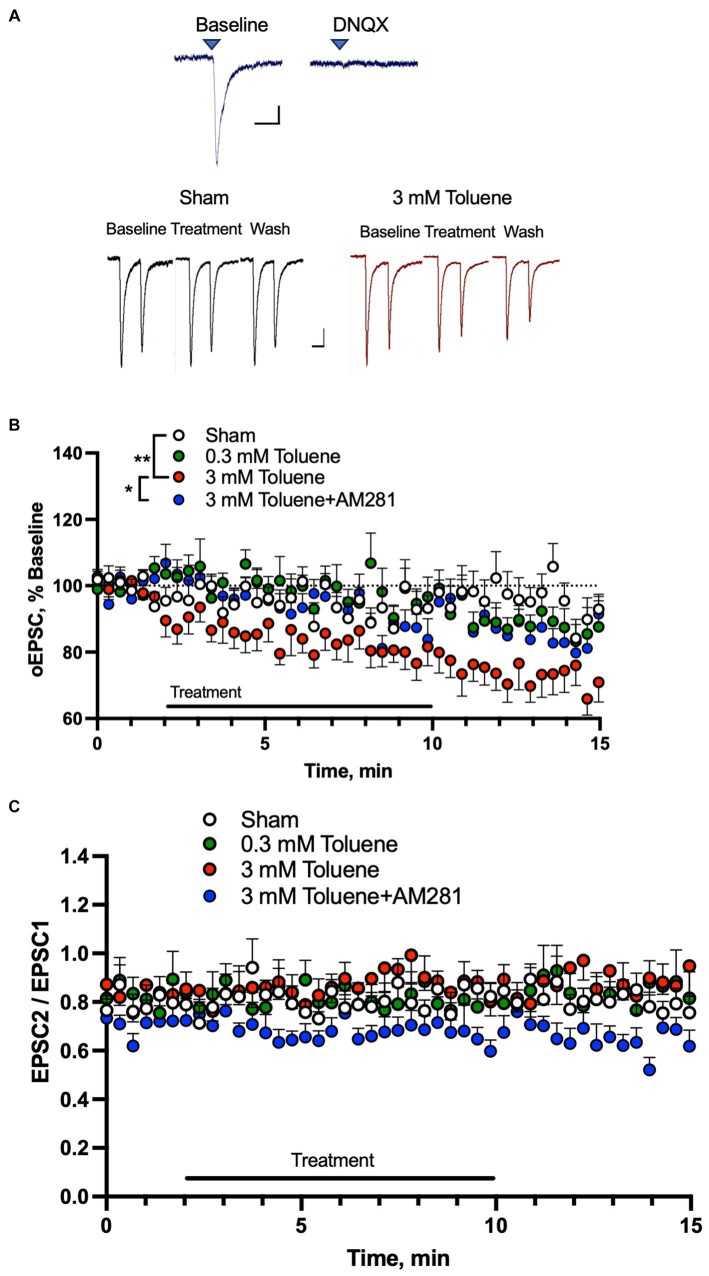
Toluene dose-dependently reduces excitatory mPFC-BLA signaling in a CB1R-dependent manner. **(A)** EPSCs in BLA principal neurons evoked by light pulses (470 nm) in slices from rats expressing channelrhodopsin-2 in mPFC terminals. Top traces show EPSCs before (left) and after (right) bath application of the AMPA receptor antagonist DNQX (10 μM). Scale bars: *x*-axis 50 msec, *y*-axis 50 pA. Bottom traces show representative AMPA-mediated EPSCs before, during and after Sham aCSF or toluene (3 mM) exposure. Scale bars: *x*-axis 100 msec, *y*-axis 50 pA. **(B)** Time course of EPSC1 before, during and after exposure to Sham aCSF, 0.3 mM toluene, 3.0 mM toluene or 3.0 mM plus 0.75 μM AM281. Symbols: (**) indicates significant difference (*p* < 0.01) between Sham aCSF and 3 mM toluene group, repeated measures mixed model with Dunnett’s post-hoc test. (*) indicates significant difference (*p* < 0.05) between 3 mM toluene and 3 mM toluene plus 0.75 μM AM281 (Sidak’s post-hoc test). **(C)** Time course of paired pulse ratio (EPSC 2/EPSC 1) before, during and after exposure to Sham aCSF, 0.3 mM toluene, 3.0 mM toluene or 3.0 mM plus 0.75 μM AM281. Data are expressed as a percent (mean + SEM) of pre-treatment baseline; aCSF Sham (*n* = 11 cells/6 animals), 0.3 mM toluene (*n* = 10 cells/6 animals), 3.0 mM toluene (*n* = 11/4 animals), and 3.0 mM toluene +0.75 μM AM 281 (*n* = 8 cells/6 animals).

Toluene disrupts mPFC-BLA dependent behavior (St Onge et al., 2012; Jenni et al., 2017; Braunscheidel et al., 2019), and this circuit also mediates alcohol withdrawal-related neurophysiology (Gioia et al., 2017; Gioia and McCool, 2017). To determine the effects of toluene on glutamatergic mPFC-BLA synapses, we expressed channelrhodopsin-2 (ChR2) in the prelimbic mPFC and recorded light-evoked (470 nm) EPSCs in BLA neurons surrounded by ChR2 expressing mPFC terminals. We verified that these responses were mediated by AMPA receptors as they were blocked by the antagonist DNQX (10 μM; Figure 3A, top traces). We then tested the effects of aCSF or toluene on BLA AMPA EPSCs using pairs of light pulses (Figure 3A, bottom traces) and each neuron was exposed to only one condition and data were then expressed as a percent of the response normalized to the 2 min baseline period. Figure 3B shows the amplitude of EPSC 1 from BLA neurons before, during and after exposure to aCSF (Sham), 0.3 mM, 3 mM toluene and 3 mM toluene applied in aCSF containing 0.75 μM of the CB1 receptor antagonist AM281. Statistical analysis of this data revealed a significant main effect of treatment (*F*_(3,36)_ = 5.47, *p* = 0.003, repeated measures mixed effects model) and post-hoc testing of the Sham versus experimental groups showed a significant difference between Sham and 3 mM toluene (Dunnett’s post-hoc, *q*_(3.50)_, *p* = 0.0036) but no difference between Sham and 0.3 mM toluene (Dunnett’s post-hoc, q_(0.048)_, *p* > 0.99) or Sham and 3 mM toluene plus 0.75 μM AM281 (Dunnett’s post-hoc, *q*_(0.71)_, *p* = 0.82). Post-hoc pairwise testing also showed a significant difference between the 3 mM toluene group and the 3 mM toluene plus 0.75 μM AM281 group (Sidak’s post-hoc, *t*_2.49_, *p* = 0.018). Figure 3C shows the paired pulse ratio (PPR expressed as EPSC2/EPSC1) before, during and after treatment with aCSF (Sham), 0.3 mM toluene or 3.0 mM toluene, or 3.0 mM toluene with 0.75 μm AM281. Statistical analysis of this data showed no significant change in the PPR over the 15 min testing period under any condition (main effect of treatment *F*_(3,36)_ = 1.71, *p* = 0.18; time × treatment *F*_(132,1575)_ = 0.96, *p* = 0.61; mixed effects model).

## Discussion

A major finding of this study is that a brief exposure to toluene produced a small (~20%) but significant increase in the excitability of principal basolateral amygdala (BLA) glutamatergic pyramidal neurons that reversed upon washout. The increase in firing during an acute *in vitro* exposure to toluene complements a previous finding showing increased c-Fos expression, a proxy for increased neuronal activity, in the BLA and other brain regions following *in vivo* exposure to toluene (Perit et al., 2012). Several components of the action potential of BLA neurons were also affected by toluene treatment including decreased latency to fire, decreased inter-spike interval, and a reduced fast after-hyperpolarization potential (AHP) and these changes likely contributed to the increase in firing during toluene exposure. Toluene-mediated increases in neuronal excitability have been reported for some but not all brain regions examined. For instance, in slice recordings, toluene increased the tonic firing of dopamine neurons within the VTA (Riegel and French, 1999; Riegel et al., 2007; Nimitvilai et al., 2016), but not outside of the ventral tegmental area (Riegel et al., 2007). In contrast, action potential firing of nucleus accumbens neurons (Beckley et al., 2016) or deep layer prelimbic mPFC neurons (Beckley and Woodward, 2011) was not affected by acute application of toluene to the slice. However, 24 h following a single *in vivo* exposure to binge-like (~10,000 ppm) levels of toluene vapor, mPFC neurons that project to the nucleus accumbens show sub-region, projection and layer-specific changes in excitability (Wayman and Woodward, 2018b). Core-projecting mPFC neurons in layer 5/6 prelimbic mPFC were hypo-excitable following the toluene exposure while those in layer 2/3 were not affected. In contrast, toluene exposure enhanced firing of core-projecting neurons in both deep and shallow layers of the infralimbic mPFC. Shell projecting neurons in the infralimbic mPFC were hypoexcitable following toluene treatment with no effect seen in shell projecting neurons from layer 2/3 infralimbic mPFC or those from the prelimbic mPFC. Twice daily exposures to toluene vapor over a 10 days period was shown to increase the excitability of deep layer prelimbic mPFC neurons although the projection specificity of those neurons was not determined (Armenta-Resendiz et al., 2018). A recent study from our laboratory examined changes in the intrinsic excitability of nucleus accumbens medium spiny neurons (MSNs) 1 day after a single exposure to 10,000 ppm toluene. Toluene exposure had no effect on current-evoked spiking of MSNs in the nucleus accumbens core but caused a leftward shift in the rheobase of those in the nucleus accumbens shell accompanied by a depolarization block of firing at higher current amplitudes (Okas et al., 2023). Using a transgenic Cre expressing line of rats, this effect was shown to be driven by changes in D2 but not D1 NAc shell neurons. Taken together, these findings reveal a surprising degree of selectively of toluene action on neural activity following *in vitro* or *in vivo* exposure to toluene that, as discussed below, may reflect differential expression of toluene targets between various neuron subtypes and circuits.

The underlying cause of the increased excitability of BLA neurons during acute exposure to toluene is not completely known. An interesting possibility is that toluene, via direct inhibition of large conductance calcium-activated potassium (BK) channels (Del Re et al., 2006) reduced the neurons relative refractory period (Sah and Faber, 2002). This explanation is consistent with observed decrease in the BK-dependent fast afterhyperpolarization potential (AHP) and decreased inter-spike interval by toluene in the present study. Beckley and colleagues also found that toluene dampens the fast component of the AHP in medium spiny neurons in the nucleus accumbens (Beckley et al., 2016), but had no effect on the AHP in mPFC neurons (Beckley and Woodward, 2011). Together with the findings of the present study, these results show that toluene’s effects on BK-mediated components of neuronal excitability are region specific possibly due to differential expression of BK channel subtypes that may contribute to their toluene sensitivity.

While the reduction in AHP and inter-spike interval is a likely explanation for driving the observed toluene-induced increases in excitability, toluene-mediated reductions in AHP have been observed in the absence of signficant changes in excitability (Beckley et al., 2016). Further, this mechanism does not appear to be responsible for the toluene-induced hyperexcitability of nucleus accumbens core-projecting prelimbic mPFC neurons (Wayman and Woodward, 2018b), although this may reflect differences between the *in vitro* and *in vivo* exposure protocols used in these studies. While some effects of toluene on action potential parameters observed in the present study would be expected to increase excitability, others are more consistent with reduced excitability including increased action potential rise time and decay. These effects could be due to toluene’s effect on voltage gated sodium channels (Cruz et al., 2003; Gauthereau et al., 2005) although it is noted that these studies were conducted with channel subtypes expressed primarily in cardiac or skeletal muscle tissue.

Despite its effect on intrinsic activity, toluene is generally regarded as a central nervous system depressant via actions on voltage-gated calcium channels (Tillar et al., 2002; Shafer et al., 2005), NMDA receptors (Cruz et al., 1998, 2000), nicotinic acetylcholine receptors (Bale et al., 2002), GABA_A_, and glycine receptors (Beckstead et al., 2000, 2001). In the present study, we found that 3 mM toluene inhibited mPFC-dependent AMPA receptor excitatory post-synaptic currents (EPSCs) in the BLA. Given *in vitro* volatility losses of 20%–25% (Cruz et al., 1998) and estimates that ~3% of inhaled toluene reaches the brain (Benignus et al., 1981), this dose equates roughly to 7,400 ppm, a concentration similar to that encountered by humans during voluntary solvent inhalation (Brouette and Anton, 2001; Bukowski, 2001). Toluene’s inhibitory effect on BLA AMPA currents progressed slowly over the course of treatment, persisted during washout and was prevented by co-application of the CB1 receptor inverse agonist AM281. These findings are consistent with the lack of a direct effect of toluene on recombinant AMPA receptors (Cruz et al., 1998) and results from previous studies from our laboratory showing that toluene-induced decreases in AMPA EPSCs in the NAc (Beckley et al., 2016) and mPFC (Beckley and Woodward, 2011) are CB1 receptor-dependent and involve release of calcium from intracellular stores linked to the synthesis of endogenous endocannabinoids. Interestingly, in the NAc study, the effect of toluene on AMPA-mediated EPSCs was restricted to D2 MSNs again highlighting a cell-type specificity of toluene action (Beckley et al., 2016). Endocannabinoid mediated inhibition of synaptic transmission is thought to occur via a presynaptic mechanism as indicated by changes in the paired pulse ratio of evoked responses and a reduction in the frequency of spontaneous and miniature synaptic events (Busquets-Garcia et al., 2018). In the present study, although the CB1R antagonist AM281 prevented the toluene inhibition of BLA AMPA-mediated EPSCs, toluene itself did not significantly alter the paired pulse ratio of optically evoked synaptic events. A previous study reported a reduction in EPSC amplitude accompanied by a small (~19%) but significant increase in the PPR of electrically evoked BLA AMPA EPSCs following exposure to the direct acting CB1R agonist WIN-55-212 (Azad et al., 2003). Similarly, ethanol induced a small increase (~9%) in the PPR of BLA AMPA EPSCs in some but not all neurons tested along with a decrease in AMPA EPSC amplitude that was blunted by CB1R antagonists (Robinson et al., 2016). Both of those studies used a shorter inter-pulse interval (50 msec) than in the present study (150 msec) that may allow better detection of small changes in PPR. In addition, there are reports of differences in paired pulse plasticity between electrically and optically evoked responses that could have contributed to differences between these studies and the present one (reviewed by Jackman et al., 2014). It is important to note that while most studies suggest that projections from mPFC to BLA are glutamatergic, there is evidence for long-range GABAergic neurons in the frontal cortex. For example, expressing ChR2 in GABergic neurons in the medial prefrontal cortex via use of the Dlxi12b-Cre mouse line revealed fibers projecting to subcortical areas including the NAc, BLA, dorsal striatum and claustrum (Lee et al., 2014). Electrophysiological recordings revealed light-evoked currents in approximately half of the NAc neurons examined and these currents were blocked by the GABA_A_ antagonist gabazine. Whether the ChR2 stimulated inward currents in BLA neurons recorded in the present study may have included those mediated by long-range GABA neurons is not known but is possible due to the lack of a GABA_A_ antagonist in the bath and use of a high chloride containing pipette solution. However, we observed that the light-evoked currents were blocked by the AMPA receptor antagonist DNQX and note that we selectively targeted the prelimbic division of the rat mPFC while Lee et al. (2014) expressed ChR2 in the anterior cingulate, prelimbic and infralimbic areas of the Dlxi12b-Cre mouse line. Nonetheless, at the present time, we can not rule out that the endocannabinoid-dependent toluene-induced suppression of light-evoked currents in BLA neurons may have been a mixture of glutamatergic and GABAergic responses.

The present study is the first to show that acute toluene impairs neural activity and synaptic signaling in the mPFC-BLA neural circuit, a pathway implicated in alcohol-seeking behavior in C57BL/6J mice (Gioia et al., 2016, 2017; Gioia and McCool, 2017). The toluene inhibition of mPFC-BLA signaling observed in the current study is interesting in light of recent work from our lab investigating risk/reward decision making following exposure of male and female rats to binge-like concentrations (6,000–10,500 ppm) of toluene vapor (Braunscheidel et al., 2019). In that study, toluene exposure produced deficits in behavioral flexibility that mimicked those observed following pharmacological inactivation of the mPFC-BLA pathway (St Onge et al., 2012; Jenni et al., 2017). This was manifested as a delayed shift in their choice behavior as the odds of receving a large/risky reward changed suggesting that a persistent CB1R-mediated suppression of mPFC or BLA excitatory signaling may have contributed to the reduction in behavioral flexibility in toluene treated animals. However, in a follow-up study, systemic or intra-mPFC infusion of CB1R antagonists failed to prevent changes in risk behavior in rats acutely exposed to toluene vapor despite producing selective effects on choice behavior and latency on their own (Braunscheidel et al., 2021). While these results do not rule out a role for toluene-induced inhibition of mPFC-BLA synaptic activity in this effect, future studies using intra-BLA infusion of CB1R antagonists or genetic manipulation of BLA CB1R signaling are needed to fully address this issue.

To our knowledge, these studies are the first to investigate the effect of the inhalant toluene on BLA neurophysiology. Using whole-cell patch clamp electrophysiology, we found that a concentration of toluene that is associated with voluntary inhalant abuse transiently increased the excitability of BLA pyramidal neurons. The increase in firing was accompanied by a significant reduction in the fast AHP potential and decreased inter-spike interval. Optical stimulation of mPFC terminals in the BLA revealed that toluene induced a CB1R-dependent decrease in AMPA-mediated excitatory signaling. These findings add to those clearly demonstrating the ability of a commonly used inhalant to alter neural activity and signaling in key addiction related brain regions.

## Data availability statement

The raw data supporting the conclusions of this article will be made available by the authors, without undue reservation.

## Ethics statement

The animal study was approved by MUSC Institutional Animal Care and Use Committee. The study was conducted in accordance with the local legislation and institutional requirements.

## Author contributions

KB: Formal analysis, Funding acquisition, Investigation, Methodology, Writing – original draft, Writing – review & editing. MO: Investigation, Methodology, Writing – review & editing. JW: Conceptualization, Formal analysis, Funding acquisition, Supervision, Writing – original draft, Writing – review & editing.
